# Reducing Exposures to Endocrine Disruptors (REED) study, a personalized at-home intervention program to reduce exposure to endocrine disrupting chemicals among a child-bearing age cohort: study protocol for a randomized controlled trial

**DOI:** 10.1186/s13063-024-08627-3

**Published:** 2024-11-25

**Authors:** Johanna R. Rochester, Carol F. Kwiatkowski, Michael Kupec Lathrop, Iva Neveux, Eric J. Daza, Joseph Grzymski, Jenna Hua

**Affiliations:** 1 Million Marker Wellness, Inc, Berkeley, California USA; 2 University of Nevada, Reno, Reno, Nevada USA; 3 The Healthy Nevada Project, Renown Health, Reno, Nevada USA

## Abstract

**Background:**

Exposures to endocrine disrupting chemicals (EDCs) have been linked to chronic diseases including breast cancer, metabolic syndrome, diabetes, and infertility. Exposure during pregnancy may have a lifelong impact on the fetus. Services are needed to allow individuals to learn about their personal EDC exposures and how to reduce them. Million Marker (MM) aims to crowdsource and scale the biomonitoring of environmental chemicals and provide actionable results to empower individuals to proactively assess, track, and reduce their EDC exposures. In previous research, we developed and tested the first mobile EDC intervention service (mail-in urine testing and exposure report-back) for its efficacy in increasing EH literacy (EHL), willingness to reduce exposures (i.e., readiness to change, RtC), and system usability. After intervention, we found increased EHL, increased RtC in women (but not men), and decreased EDC exposure. However, some participants did not increase their RtC and had difficulty carrying out the intervention on their own. The reasons for these less optimal results were the difficulty in the EHL subject matter—participants still felt ill-prepared to apply their knowledge to making healthier lifestyle changes. Therefore, in this study, we will address these perceived limitations.

**Methods:**

We will test a self-directed online interactive curriculum with live counseling sessions and individualized support modeled after the highly effective Diabetes Prevention Program (DPP). Recruiting from the Healthy Nevada Project (HNP), one of the largest population health cohorts in the world, we test the effectiveness of our EDC-specific online intervention curriculum via EHL and RtC surveys and determine changes in EDC exposure before and after intervention in a randomized controlled trial. We will also test for common clinical biomarkers via a commercially available at-home test (Siphox). We will recruit and randomize 300 women and 300 men of reproductive age (total *n*=600) from HNP. Our target population is men and women of reproductive age (18–44 years old).

**Discussion:**

At the conclusion of this project, we will be well-positioned to scale our services to clinics and the general public, with the eventual aims of FDA approval, insurance coverage, and incorporation into routine clinical care.

**Supplementary Information:**

The online version contains supplementary material available at 10.1186/s13063-024-08627-3.

## Background

### The exposome, endocrine-disrupting chemicals, and health

The exposome encompasses the totality of environmental exposures across one’s lifespan [1, 2]. It includes exposures from the food and water, air and household and personal products, as well as social capital, stress, and other contextual elements [3]. The exposome works in tandem with the genome to determine our health and physiological state [1]. Many everyday products contain endocrine-disrupting chemicals (EDCs), which can alter the normal endocrine function of biological systems. Due to their ubiquitousness, impacts on the environment, wildlife, and human health, EDCs have become a major public health concern [4]. Data from the National Health and Nutrition Examination Survey (NHANES) has shown that more than 90% of US adults have detectable levels of common EDCs, such as bisphenol A (BPA) and phthalates, in their urine [5, 6]. Exposures to EDCs have been linked to chronic diseases in both men and women, including breast cancer, [7, 8] obesity, [9–11] metabolic syndrome, [12] diabetes, [13] and infertility [14, 15]. EDC exposures are particularly concerning for reproductive-aged women. Women are the primary consumers of many personal care products, [6] and exposures during pregnancy predispose the fetus to adverse health effects later in life, [16] including cancers, IQ loss, neurotoxicity, attention-deficit hyperactivity disorder, childhood obesity, adult obesity, Type 2 diabetes, cardiovascular and metabolic disease, genital defects, infertility, pregnancy complications, and mortality from reduced testosterone [4, 7, 9–11, 16–24].

### Need for EDC intervention

Endocrine-disrupting chemicals (EDCs), including bisphenols, phthalates, parabens, and oxybenzone, are present in over 90% of the population at any one time, [5, 6] and have been linked with numerous health effects in laboratory and human studies, including breast cancer, [7, 8] obesity, [9–11] metabolic syndrome, [12] diabetes, [13] and infertility, [14, 15] and effects on the fetus that can manifest later in life, including cancers, IQ loss, neurotoxicity, attention-deficit hyperactivity disorder, childhood obesity, adult obesity, type 2 diabetes, cardiovascular and metabolic disease, genital defects, infertility, pregnancy complications, and mortality from reduced testosterone [4, 7, 9–11, 16–25].

BPA is used to make polycarbonate plastic and epoxy resins and is found in numerous consumer products (e.g. thermal receipts, plastic foodware, can linings). BPA is a known hormone disruptor and has been linked to numerous health effects in laboratory animals [23] and chronic diseases and disorders in humans, including infertility, cardiovascular and metabolic disease, asthma, and child development [16]. It has also been deemed a mammary carcinogen [24]. Due to these health concerns, BPA has been increasingly replaced with other bisphenols (BPs), which are structurally or functionally similar to BPA, (such as BPS and BPF) and share many of the same health concerns as BPA [26–29]. Phthalates are used as to make plastic flexible and are added to fragrances to disperse scent [30]. Phthalates are also hormone disruptors; they have long been known for antiandrogenic effects (i.e. decreased anogenital distance in infant boys) [31–33]. Phthalate exposure is also associated with infertility, type 2 diabetes, asthma, and other health problems [34]. Parabens are a group of chemicals widely used as antimicrobial preservatives in personal care products and packaged food. Parabens are hormone disruptors, showing estrogenic and antiandrogenic activity, as well as blocking aromatase. Exposure has been linked to adrenal and thyroid disruption, metabolic changes, developmental-behavioral changes in animals, [35] and reduced fertility in humans [36]. Oxybenzone is a UV filter that is found in many sunscreens, hair products, cosmetics, and lotions [37, 38]. Oxybenzone is an EDC—it has been shown to be proliferative in estrogen-responsive cancer cell assays, [38, 39] as well as promoting the growth of androgen-dependent prostate cancer cells [40].

These EDCs have relatively short half-lives in the body (i.e. 6 hours to 3 days) [41–43]. Therefore, the fact that most of the population is exposed at any one time [5, 6] indicates ubiquitous and constant exposure sources. However, the rapid elimination from the body also allows for reduction of internal exposure with removal or avoidance of the exposure sources from daily life. Thus, interventions aimed at identifying exposures (through internal and external assessments) and removing them (via personalized recommendations based on exposure levels and lifestyle habits), have the potential to greatly reduce transient EDC exposures, as demonstrated in our previous work [44].

### Previous work

In previous work, [44] participants in the Healthy Nevada Project completed EHL and readiness to change surveys before (*n* = 424) and after (*n *= 174) an EDC exposure report-back intervention (measured via the MM mail-in testing kit). Report-back of results included urinary levels, information about health effects, sources of exposure, and personalized recommendations to reduce exposure. Participants ranged in age from 18-61, were primarily women (75%), and white (79%). EHL was generally very high at baseline. Higher EHL knowledge was shown among older participants (*p* = 0.02) and those self-rated in poorer health (*p *= 0.007). EHL behaviors increased after report-back (*p *= 0.003).

For readiness to change, 72% were already or planning to change their behaviors. Women were generally in earlier stages than men (p=0.071), but post-intervention, women increased their readiness (p=0.053), while men decreased (p=0.007). When asked what challenges they faced in reducing exposure, 79% cited not knowing what to do. This dropped to 35% after report-back. Participants with higher propylparaben were younger (p=0.03), women and participants who rated themselves in better health had higher levels of some phthalates (p=0.02-0.003 and p=0.001-0.003 respectively). After report-back, monobutyl phthalate decreased among the 55 participants who submitted a second urine test (p<0.001). Of the 50 participants who completed final surveys, 50% reported now using non-toxic personal products, 44% were using non-toxic household products, 20% dined out less, 32% ate less packaged food, 40% used less plastic, and 48% read product labels more.

The report-back intervention was successful as evidenced by increased EHL behaviors, increased readiness to change among women, a 44% reduction in the percentage of participants not knowing how to decrease exposure, a high percentage of participants reporting behavior changes to reduce exposure, and a decrease in monobutyl phthalate. An EHL questionnaire more sensitive to chemical exposures would help differentiate high and low literacy. Therefore, in the current study, a more sensitive EDC EHL survey will be employed. And although we observed improvements in EHL and RtC after report-back, more intensive EHL education could further improve knowledge and behavior, leading to reductions in EDC exposure. Thus, in this proposal, we aim to test our newly developed EDC EHL curriculum to encourage even more EDC reduction and EHL improvement.

### EDCs and clinical biomarkers

In addition to behavioral changes of individuals and measured exposure metabolite changes, we will also test a subset of study participants for changes in clinical biomarkers that could indicate improved health. EDCs have been shown to be linked to many adverse health outcomes, including cardiovascular and metabolic disease, diabetes, low hormone levels/hormone imbalance (including thyroid problems), infertility, cancers, and inflammation [16, 45]. However, the lack of health/clinical biomarker assessments for EDC-intervention studies is a major data gap in an already limited research area. This is also reflected in our recent customer discovery interviews with physicians/clinics, employee wellness programs, and major insurers. These stakeholders require information on the changes to known clinical biomarkers (i.e. a demonstration of the effectiveness of our testing and education intervention program) in order to acquire and market our service. Showing that our intervention to reduce EDC exposures can improve clinical biomarkers will significantly increase our products’ market value and commercialization potential as well as pave the way for FDA approval and insurance/health savings account coverage for individuals.

### Report-back and curriculum

Numerous studies have demonstrated that avoidance and lifestyle behavioral change are the most effective ways to reduce EDCs, particularly non-persistent EDCs such as BPA, phthalates, and parabens [46]. For example, reducing canned food/drink consumption and avoiding touching receipts are associated with reduced BPA and BPA alternatives exposures, and using fragrance-free personal care products is associated with lower phthalate exposure [46, 47]. Such evidence-based behavioral modification strategies have been developed and provided to our Phase I participants and regular Million Marker customers in their personalized Detect & Detox Kit reports [48]. Despite this effort, we learned in Phase I that participants required more education, support, and “handholding.” In a 2022 review [47] on interventions on reducing exposure to EDCs in human health care context, the authors only found 12 intervention studies between 2011-2022 globally, with sample sizes ranging from 20-218 participants and intervention durations of 3 days to 6 months. Unlike our Phase I study, none of the studies provided report-back to the participants. We have also recruited over 400 participants, which made us the largest intervention study to date. The authors pointed out that only four studies provided their participants with education and four let their participants choose alternative goods or foods themselves, which suggested the lack of self-directed behavior change and real-world application and generalizability. Most of the studies intervened in one aspect (i.e., dietary modification vs. using alternative household or personal care products) of participants’ lifestyle behavior rather than all potential sources of EDCs, and it was unclear whether the short-term behavior changes could be sustained over a long period of time or throughout the life course. Lastly, the review indicated the need for randomized controlled trials and participant-centered studies promoting active participation and practices. Therefore, insights derived from previous research and recommendations from the latest scientific publications indicate the need to enhance our existing service with a participant-driven education behavioral intervention program that builds behavioral change knowledge and skills that we can put to test through a randomized longitudinal study.

Sustained lifestyle and health behavioral change requires knowledge and skills that are based on the science of behavior change [49]. Behavioral counseling programs with consistent, regular interaction with a health coach combined with education, support and skill building have been proven effective for reducing chronic disease risks [50]. Given the success of the DPP and Omada Health (digitization of the DPP) in achieving behavioral and clinical outcome goals, as well as their proven framework of translatability (including training, intervention delivery, support and evaluation) [51] and business model, it is prudent to model our education behavioral intervention program after DPP and Omada Health.

The DPP has proven to be highly effective at curbing diabetes through achieving lifestyle goals of a minimum 7% weight loss/weight maintenance and a minimum of 150 minutes of physical activity similar in intensity to brisk walking [52]. The success of DPP was attributed to other key features, including 1) individual lifestyle coaches; 2) frequent contact with participants; 3) a structured 16-session core-curriculum that taught behavioral self-management strategies for weight loss and physical activity; 4) physical activity sessions; 5) flexible maintenance intervention combining group and individual approaches and motivational campaigns; 6) individualization through a diabetes toolbox of adherence strategies; 7) tailoring of materials and strategies to address ethnic diversity, and 8) an extensive network of training, feedback, and clinical support [52].

Given the flexibility, accessibility, convenience, and scalability of digital health and mobile interventions, [50, 53] Omada Health pioneered the translation of DPP into digital format and demonstrated its long-term effectiveness [54]. The Omada’s program consists of a weekly interactive behavior change curriculum focused on weight loss and physical activity, digital tracking tools, personalized health coaching, and online small group support. The lessons include reading content, interactive games or exercise, and written reflections and goal-setting activities [50].

Leveraging the insights, key ingredients and program structure of DPP and Omada’s digital DPP program, our self-directed online interactive education curriculum will consist of the following 1) evidence-based 8-lesson curriculum covering topics related to EDCs (basic science of EDCs, health impact, exposure sources, etc.), see details in Table 1; 2) take-home handouts, practical tip sheets and checklists to facilitate behavior reinforcement; 3) live coaching sessions at the end of each lesson; 4) individualized support through online messaging, text messaging, phone and video calls; 5) community support through an online forum, and 6) personalized exposure biomarker testing and report-back with tailored behavioral and product recommendations. We will put strong emphasis on what people can control and small steps to reduce harmful exposures. Our curriculum and interventions will be grounded in social cognition theory, where behavioral change begins with individuals’ perception of their behavior on health, [55] as well as Theory of Planned Behavior, where we will promote a sense of self-efficacy and utilize techniques such as goal setting, feedback, self-regulation and social support to achieve and sustain EDC-reduction behaviors [56–58].

**Table 1:** Example topics covered in Million Marker’s 8-lesson curriculum

Class	Topics Covered	Description
1	Course orientation, introduction to environmental health and EDCs	● Overview of the endocrine system ● Overview of environmental health and EDCs ● Persistent vs. transient chemicals ● Acute vs. chronic exposures ● Chemical absorption, metabolism, distribution and excretion ● EDCs and fertility, fetus/child development ● **Reflective journal promp**t: take a look around your room and determine some primary EDC exposures
2	Chemicals in personal care products, beauty products, cosmetics	● Soap, hair care, skin care, oral care ● Feminine hygiene and intimate products ● Fragrance/perfume/parfum, makeups ● How to read ingredient labels ● **Reflective journal prompt:** what’s on your plan of action to reduce EDC exposures?
3	Exposures from kitchen, bedroom, living room, bathroom	● Kitchen: dish detergent, all-purpose cleaners, sponge, wipes/antibacterial products ● Living rooms/bedrooms: scented plug-ins/candles/air fresheners, dust/mold ● Bathroom: shower curtain, toilet cleaner, mold/mildew cleaner ● **Reflective journal prompt**: what are some cleaning products that you need or don’t need?
4	Exposures around the house and the house itself	● Textiles: clothing, bedding, rugs ● Furniture, floors ● **Reflective journal prompt**: what are some upcoming big-ticket purchases? What would you consider when making such purchases?
5	Exposures from food, water, and medications	● Contamination and exposures in food ● Water: municipal water and well water, water testing, water filters ● Supplements/medications: capsule coating, medical supplies and dental sealants ● **Reflective journal prompt**: How many foods that you eat in a day that are processed vs whole foods?
6	Shopping for, cooking, and ordering Foods	● How to read ingredient labels ● How to shop ● Food packaging, cookware, food storage ● **Reflective journal prompt**: How often do you cook at home?
7	Clothing, electronics, and cars	● Jewelry, clothing, and bedding ● Researching clothing ● Electronics, cars, and hobbies ● **Reflective journal prompt**: what are ways you can reduce your exposures that require some planning?
8	Course wrap-up and review	● Course review and highlight major takeaways ● **Reflective journal prompt**- do you have any plans for the next 6 months? 12 months?

**Table 2:** SPIRIT Guideline Checklist

Section/Item	Reporting Item	Description	Page and Line Number	Reason if not applicable
Title	#1	Descriptive title identifying the study design, population, interventions, and, if applicable, trial acronym	p. 1, 1-3	
Trial registration	#2a	Trial identifier and registry name. If not yet registered, name of intended registry	Clinical Trials, NCT06450951 (https://www.clinicaltrials.gov/)	
Trial registration: data set	#2b	All items from the World Health Organization Trial Registration Data Set	All items included in the Methods and Intervention, p. 9-21	
Protocol version	#3	Date and version identifier	p. 23, 545-547	
Funding	#4	Sources and types of financial, material, and other support	p. 26, 612-615	
Roles and responsibilities: contributorship	#5a	Names, affiliations, and roles of protocol contributors	p. 26, 617-621	
Roles and responsibilities: sponsor contact information	#5b	Name and contact information for the trial sponsor	p. 1, 11	
Roles and responsibilities: sponsor and funder	#5c	Role of study sponsor and funders, if any, in study design; collection, management, analysis, and interpretation of data; writing of the report; and the decision to submit the report for publication, including whether they will have ultimate authority over any of these activities	p. 26, 614-615	
Roles and responsibilities: committees	#5d	Composition, roles, and responsibilities of the coordinating centre, steering committee, endpoint adjudication committee, data management team, and other individuals or groups overseeing the trial, if applicable (see Item 21a for data monitoring committee)	p. 26, 612-621	
**Introduction**				
Background and rationale	#6a	Description of research question and justification for undertaking the trial, including summary of relevant studies (published and unpublished) examining benefits and harms for each intervention	p. 3-8	
Background and rationale: choice of comparators	#6b	Explanation for choice of comparators	p. 9, 201-204	
Objectives	#7	Specific objectives or hypotheses	p. 9, 201-204	
Trial design	#8	Description of trial design including type of trial (eg, parallel group, crossover, factorial, single group), allocation ratio, and framework (eg, superiority, equivalence, non-inferiority, exploratory)	p. 4-5, 204-207	
**Methods: Participants, interventions, and outcomes**				
Study setting	#9	Description of study settings (eg, community clinic, academic hospital) and list of countries where data will be collected. Reference to where list of study sites can be obtained	p. 14, 218-219	
Eligibility criteria	#10	Inclusion and exclusion criteria for participants. If applicable, eligibility criteria for study centres and individuals who will perform the interventions (eg, surgeons, psychotherapists)	p. 9, 221-224	
Interventions: description	#11	Interventions for each group with sufficient detail to allow replication, including how and when they will be administered	p. 11-16	
Interventions: modifications	#11b	Criteria for discontinuing or modifying allocated interventions for a given trial participant (eg, drug dose change in response to harms, participant request, or improving / worsening disease)	p. 10, 249-250	
Interventions: adherance	#11c	Strategies to improve adherence to intervention protocols, and any procedures for monitoring adherence (eg, drug tablet return; laboratory tests)	p. 10, 249-250; p. 13-14, 285-302	
Interventions: concomitant care	#11d	Relevant concomitant care and interventions that are permitted or prohibited during the trial		None, not monitoring any other care/interventions of participants
Outcomes	#12	Primary, secondary, and other outcomes, including the specific measurement variable (eg, systolic blood pressure), analysis metric (eg, change from baseline, final value, time to event), method of aggregation (eg, median, proportion), and time point for each outcome. Explanation of the clinical relevance of chosen efficacy and harm outcomes is strongly recommended	p. 15-17, 322-376	
Participant timeline	#13	Time schedule of enrolment, interventions (including any run-ins and washouts), assessments, and visits for participants. A schematic diagram is highly recommended (see Figure)	p. 14-15, 304-320; Fig. 1	
Sample size	#14	Estimated number of participants needed to achieve study objectives and how it was determined, including clinical and statistical assumptions supporting any sample size calculations	p. 11, 252-255; p. 16-17, 364-371	
Recruitment	#15	Strategies for achieving adequate participant enrolment to reach target sample size	p. 9-10, 218-250	
**Methods: Assignment of interventions (for controlled trials)**				
Allocation: sequence generation	#16a	Method of generating the allocation sequence (eg, computer-generated random numbers), and list of any factors for stratification. To reduce predictability of a random sequence, details of any planned restriction (eg, blocking) should be provided in a separate document that is unavailable to those who enrol participants or assign interventions	p. 14, 305-306	
Allocation concealment mechanism	#16b	Mechanism of implementing the allocation sequence (eg, central telephone; sequentially numbered, opaque, sealed envelopes), describing any steps to conceal the sequence until interventions are assigned	P. 9, 207-208	
Allocation: implementation	#16c	Who will generate the allocation sequence, who will enrol participants, and who will assign participants to interventions	p. 9, 209-210	
Blinding (masking)	#17a	Who will be blinded after assignment to interventions (eg, trial participants, care providers, outcome assessors, data analysts), and how	p. 9, 213-215	
Blinding (masking): emergency unblinding	#17b	If blinded, circumstances under which unblinding is permissible, and procedure for revealing a participant’s allocated intervention during the trial		Not applicable, since this is not a drug trial, there is no reason to reveal the intervention status during the trail
**Methods: Data collection, management, and analysis**				
Data collection plan	#18a	Plans for assessment and collection of outcome, baseline, and other trial data, including any related processes to promote data quality (eg, duplicate measurements, training of assessors) and a description of study instruments (eg, questionnaires, laboratory tests) along with their reliability and validity, if known. Reference to where data collection forms can be found, if not in the protocol	p. 13, 291-294; p. 15, 323-337; data collection forms are available upon request of the authors	
Data collection plan: retention	#18b	Plans to promote participant retention and complete follow-up, including list of any outcome data to be collected for participants who discontinue or deviate from intervention protocols	p. 10, 249-250; p. 13-14, 286-302	
Data management	#19	Plans for data entry, coding, security, and storage, including any related processes to promote data quality (eg, double data entry; range checks for data values). Reference to where details of data management procedures can be found, if not in the protocol	p. 17-19, 379-433; p. 20, 459-467	
Statistics: outcomes	#20a	Statistical methods for analysing primary and secondary outcomes. Reference to where other details of the statistical analysis plan can be found, if not in the protocol	p. 19-20, 435-457, Appendix 2	
Statistics: additional analyses	#20b	Methods for any additional analyses (eg, subgroup and adjusted analyses)	p. 19-20, 435-457, Appendix 2	
Statistics: analysis population and missing data	#20c	Definition of analysis population relating to protocol non-adherence (eg, as randomised analysis), and any statistical methods to handle missing data (eg, multiple imputation)	Appendix 2	
**Methods: Monitoring**				
Data monitoring: formal committee	#21a	Composition of data monitoring committee (DMC); summary of its role and reporting structure; statement of whether it is independent from the sponsor and competing interests; and reference to where further details about its charter can be found, if not in the protocol. Alternatively, an explanation of why a DMC is not needed	p. 20, 459-467	
Data monitoring: interim analysis	#21b	Description of any interim analyses and stopping guidelines, including who will have access to these interim results and make the final decision to terminate the trial		None, we will not be conducting interim analyses.
Harms	#22	Plans for collecting, assessing, reporting, and managing solicited and spontaneously reported adverse events and other unintended effects of trial interventions or trial conduct	p. 10, 238-249, Appendix 1	
Auditing	#23	Frequency and procedures for auditing trial conduct, if any, and whether the process will be independent from investigators and the sponsor	p. 21, 496-498	
**Ethics and dissemination**				
Research ethics approval	#24	Plans for seeking research ethics committee / institutional review board (REC / IRB) approval	p. 24-25, 582-590	
Protocol amendments	#25	Plans for communicating important protocol modifications (eg, changes to eligibility criteria, outcomes, analyses) to relevant parties (eg, investigators, REC / IRBs, trial participants, trial registries, journals, regulators)	p. 25, 587-588	
Consent or assent	#26a	Who will obtain informed consent or assent from potential trial participants or authorised surrogates, and how (see Item 32)	p. 9-10, 224-236, Appendix 1	
Consent or assent: ancillary studies	#26b	Additional consent provisions for collection and use of participant data and biological specimens in ancillary studies, if applicable	p. 9-10, 224-236, Appendix 1	
Confidentiality	#27	How personal information about potential and enrolled participants will be collected, shared, and maintained in order to protect confidentiality before, during, and after the trial	p. 20, 465-467	
Declaration of interests	#28	Financial and other competing interests for principal investigators for the overall trial and each study site	p. 26, 610-611	
Data access	#29	Statement of who will have access to the final trial dataset, and disclosure of contractual agreements that limit such access for investigators	p. 20, 465	
Ancillary and post trial care	#30	Provisions, if any, for ancillary and post-trial care, and for compensation to those who suffer harm from trial participation	p. 14, 300-302	
Dissemination policy: trial results	#31a	Plans for investigators and sponsor to communicate trial results to participants, healthcare professionals, the public, and other relevant groups (eg, via publication, reporting in results databases, or other data sharing arrangements), including any publication restrictions	p. 25, 597-607	
Dissemination policy: authorship	#31b	Authorship eligibility guidelines and any intended use of professional writers	p. 25, 597-607	
Dissemination policy: reproducible research	#31c	Plans, if any, for granting public access to the full protocol, participant-level dataset, and statistical code	p. 25, 597-607	
**Appendices**				
Informed consent materials	#32	Model consent form and other related documentation given to participants and authorised surrogates	Appendix 1	
Biological specimens	#33	Plans for collection, laboratory evaluation, and storage of biological specimens for genetic or molecular analysis in the current trial and for future use in ancillary studies, if applicable	Appendix 1	

## Methods

### Study design

This study is an ongoing collaboration between Million Marker and the Healthy Nevada Project (MM-HNP Study). We will test and compare the effectiveness of 1) EDC exposure report-back (control), and  2) a self-directed online interactive curriculum of EDC EHL material, with access to live coaches and an online forum (intervention). We hypothesize that the self-directed online interactive curriculum with personalized and community support will be more effective (superior) than EDC report-back alone at reducing EDC exposures (behavior change and metabolite concentrations), as well as increasing EDC-specific EHL, readiness to reduce exposures, and well-being. These two arms will be tested in a longitudinal EDC randomized control trial (n=600). Participants will be allocated to intervention groups by the MM team via block randomization, using random-number generation software. The HNP team will generate the allocation sequence and enroll participants. Outcomes for this aim will measure surveyed changes pre- post-interventions in EDC EHL, including EDC-specific knowledge/behavior/attitudes, RtC, well-being, and urine EDC metabolite concentrations. Additionally, we will test a subset of individuals from each intervention arm (*N* = 150 from each group, for a total of *N* = 300) for clinical biomarkers using a mail-in blood card test. Outcome assessors and data analysts will be blinded to the intervention status of the participants. Additionally, participants will not be aware of their intervention status (i.e. what the other group undergoes).

### Participants

Participants will be recruited from HNP, which has collected the health and genetic information of over 50,000 participants [59]. HNP staff will recruit previously enrolled participants (who have consented to be re-contacted) through their email listserv.Our target population is adult men and women of reproductive age (18-44 years old). We expect to enroll approximately 30% racial/ethnic minorities overall. The eligibility criteria include the following: a) ages 18-44 years; b) in good health, not pregnant, free from diabetes or known kidney disease or cancer (these conditions may interfere with EDC metabolism); c) able to understand written and spoken English; and d) willing to complete all study assessments. HNP staffwill obtain onlineinformed consent from all participants and randomly assign them to either the intervention or control group of the trial.The consent form includes the following information: “This study is being conducted by Renown Institute for Health Innovation and Million Marker, a private company, as a study to demonstrate the ability to obtain important exposome data using urine samples. Results from this study may be used to apply for future funding through research grants or sponsor funded studies. As such, each entity may benefit financially if future funding is secured, however, researchers' direct compensation is not dependent on the outcomes of this research. This disclosure is made so you can decide if this relationship will affect your willingness to participate in this study. Renown Institute for Health Innovation is providing their scientific expertise and research staff to conduct this study. Million Marker is contributing the urine kits, data collection via phone app, results, and staff resources, including Jenna Hua's salary, to conduct this study. Ms. Hua is the Founder and CEO of Million Marker and is a member of the research study only involved in aggregated data analysis and research publications as a result of the study.”

Participants will be informed of any possible adverse outcomes, with the following statement: “Your participation in this study is non-invasive and there is no risk from providing a urine sample. However, there are risks involved in having your exposure information analyzed and in sharing your exposure information. Your exposure data may reveal that you have high exposure to certain chemicals, such as endocrine disrupting chemicals (EDCs), which may negatively impact your health. You may choose to seek additional healthcare advice or treatment, however any medical advice or treatment that you seek based on your results will be your financial responsibility. As with any database, despite RIHI and MM implementing rigorous privacy and security measures to protect the privacy of your information, there is always a chance that your exposure data, health information, survey responses, and/or personally identifying information may be stolen in the event of a security breach. Although RIHI and MM cannot provide a 100% guarantee that your data will be safe, they have strong policies and procedures in place to minimize the possibility of a breach. In addition to the risks noted above, there may be additional risks to participation that are currently unforeseeable.” Participants can leave the study at any time and will receive monetary compensation for completing study steps.

### Sample size

We will recruit and randomize 600 participants (1:1 male to female). According to previously published research, this has the power to detect estimated effect sizes for 13 endpoints (9 metabolites and 3 survey analyses), [6, 44, 56, 60, 61] from a sample of *n* = 150 participants per arm (i.e., after 50% attrition down from the original *n* = 300 per arm).

## Intervention

### The Millon Marker (MM) test kit and report-back

The MM EDC testing panel consists of 13 metabolites, assessing exposure to EDCs that are commonly found in household and personal products. These chemicals are found in over 90% of the US population (at any one time) [5, 15, 21, 22, 62].

Consented participants will be mailed the MM *Detect and Detox* kit. Upon receipt, participants in the intervention group will 1) engage in a Zoom call with a MM coach to complete a comprehensive 24-hour food/product Lifestyle Audit the day before their urine sample collection, 2) collect first void urine sample the next morning, 3) send back samples via FedEx, 4) receive results as well as personalized recommendations for products and lifestyle changes to reduce EDC exposures, 5) follow the recommendations and make changes to reduce exposures, 7) test again after 3 months. Participants in the control group will complete Steps 2-7, and will receive a report-back with more generalized recommendations. Participants will be guided through this journey from our online instructions, online video tutorials, extensive educational materials, and email communication.

### The endocrine-disrupting chemical (EDC) environmental health literacy (EHL) curriculum

Our 8-week education curriculum and intervention program is modeled after the Diabetes Prevention Program (DPP) [52] and Omada Health (digital DPP) [63] with the goals of building EDC-related behavioral knowledge and skills for sustained behavior change to minimize exposures. This intervention is intended to help users to 1) better understand their MM Test Kit Exposure Report results, 2) learn about various sources of toxic chemical exposures, 3) learn ways to reduce EDC exposures in various environments, 4) learn how to build behavioral skills to implement and maintain lifestyle changes practically, and 5) feel a sense of community, accomplishment, and accountability. The curriculum is self-directed, with participants required to complete at least 6 of the 8 classes within an 8-week period. See Table 1 for the course overview.

### Online coaching and community forum

For the intervention group, MM will host weekly live coaching sessions (scheduled via Calendly and delivered via Zoom) and individual support (scheduled via Calendly for phone or Zoom calls or text messaging without prior scheduling). The live “office hour”-type coaching sessions will be hosted weekly by trained *Detect and Detox* coaches employed by MM. Participants can drop in at times that are convenient for them. A minimum of 3 sessions will be required. During the sessions, coaches will facilitate group discussions about the course curriculum and answer any questions participants may have during their learning process. The coaches have a minimum of a bachelor’s degree and additional training in environmental medicine.  As we scale, we will also develop standardized coach training manuals to train more coaches. Sessions will be spot-checked to maintain the quality of coaching. Additionally, 10% of the coaching sessions will be recorded and examined for quality control. Participants are also able to email, text and schedule individual phone or video calls with coaches. Coaches will also proactively reach out to participants to support their learning and behavior change progress. This highly engaged model will allow us to support participants throughout their detox journey and provide a high-quality intervention experience. The intervention group will also have access to a MM-moderated online forum (e.g. a private Facebook group), where they can ask questions, share experiences, and engage with the study population community [64, 65]. All participants will retain access to the MM team via email after the trial for any additional questions or follow-up guidance as they continue to implement changes to reduce exposures.

### Intervention design

**Fig. 1:**
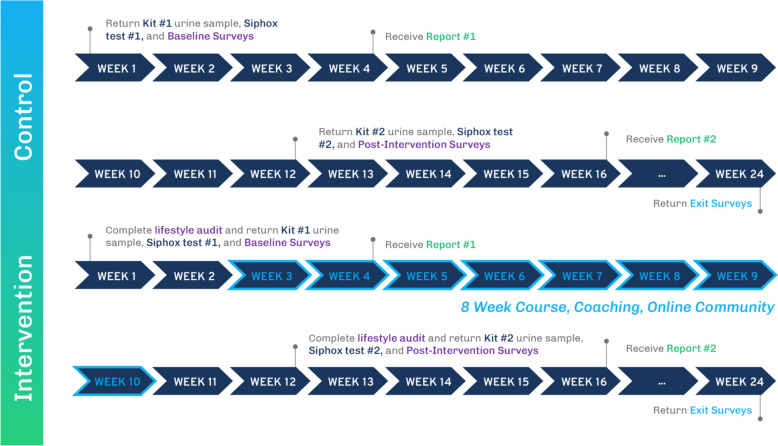
Timeline of study events for control and intervention groups

Upon consenting and randomization, all participants will be sent baseline surveys and test kits (for baseline urinary exposure levels, and, for a subset, clinical biomarkers). The control group (n = 300) will complete post-test surveys and use the second kits approximately 3 months after the baseline assessment, while the intervention group (n = 300) will complete a Lifestyle Audit and engage in the 8-week curriculum described above. The intervention group will submit the kits 2 weeks after completion of the curriculum (i.e. 3 months after the baseline test). See Fig. 1 for the control vs. intervention group study events.

Participants will be able to view the results of their Lifestyle Audit online, as well as their test results and reports. These tools will also help participants to self-monitor their behaviors and exposure reduction progress. All participants (control and intervention) will receive a report back of their urinary exposure levels. The control group will receive a list of general recommendations for exposure reduction based on their EDC exposure levels,while the intervention group will receive a more personalized exposure reduction plan based on the information collected in their Lifestyle Audit.

### Surveys, study measurements and secondary outcomes

EDC levels will be measured at baseline and 3 months. Participants' age, level of education, income, BMI, self-assessed health status, smoking status, alcohol intake, exposure to other toxic substances (e.g. dangerous chemicals, radiation), will be collected via questionnaires after recruitment to the study. We will measure participants’ environmental health literacy (EHL), readiness to change (i.e. readiness to reduce exposures, RtC), and well-being (general health, social support, financial well-being, sleep, stress, and physical activity) at baseline, 3 months (post-intervention surveys), and 6 months (exit surveys) after baseline (see Fig. 1). We will also assess usability and participant satisfaction 3 months after baseline (post-intervention). The EHL survey was adapted from the framework of the “general environmental health scale” section of the survey by Lichtveld et al. [66] which was validated in two geographic areas via exploratory and confirmatory factor analysis. Questions include those regarding individuals’ environmental health knowledge (e.g. “Household dust contains harmful chemicals”), attitudes (e.g. “I am concerned about chemicals I am exposed to through products I buy”), and behaviors (e.g. “I take steps to avoid inhaling cleaning products”). We adapted the RtC survey based on the Transtheoretical Model of Health Behavior Change [67] in which we ask about current efforts and interest in limiting exposure to harmful chemicals. EHL and RtC will be assessed to determine their association with EDC levels, as well as the effect of the intervention on EHL and RtC.

We will also measure participants’ well-being via relevant questions from three validated surveys. General health and sleep will be assessed using questions from the Behavioral Risk Factor Surveillance System; [68] stress will be measured via the Perceived Stress Scale (PSS), [69] and physical activity will be assessed using the International Physical Activity Questionnaire [70, 71]. These secondary health outcomes will be measured at baseline, 3-month and 6-months later to assess changes.

### Clinical biomarker testing

In addition to reducing EDC exposure via report-back/intervention programs, we will also assess clinical endpoints which may be improved by the reduction of EDC exposures as well as our lifestyle behavioral intervention program. To do this, we have partnered with SiPhox, a direct-to-consumer health and disease biomarker testing company [72]. SiPhox uses blood spot technologies to cost-effectively test a wide range of biomarkers involved in cardiovascular diseases and inflammation (apolipoprotein AI (Apo-AI), apolipoprotein B (ApoB), high-sensitivity C-reactive protein (hs-CRP), high-density lipoprotein (HDL), low-density lipoprotein (LDL), triglycerides (TG)), metabolic disease and diabetes (hemoglobin A1C (HbA1c), insulin), hormones and fertility (estradiol, testosterone, free testosterone, prostate-specific antigen, follicle-stimulating hormone (FSH), luteinizing hormone (LH), prolactin, sex-hormone binding globulin (SHBG), anti-Mullerian hormone, thyroid-stimulating hormone (TSH)), stress (cortisol, DHEA), and cancer (hs-CRP, prostate-specific antigen). These biomarkers can indicate risk for disease as well as the extent of ongoing disease states. Changes in these biomarkers can indicate improvement in health and reduced risk for disease. For example, high levels of high-sensitivity C-reactive protein (hs-CRP) indicates chronic inflammation and contributes to many diseases, including cancer, heart disease, lung disease, chronic obstructive pulmonary disease, chronic kidney disease, Alzheimer’s disease, diabetes, obesity, and high blood pressure. Thus reducing hs-CRP can reduce the risk for these diseases.^3^ Similarly, changes in hormone levels, such as increased testosterone, can indicate increased fertility and male reproductive health [73, 74].

We will test 150 individuals in each treatment group (i.e. report-back only group (control) vs. the intervention group (treatment)) for a total of N=300. Sample size estimates were based on a systematic review and meta-analysis of low-fat diets and testosterone measurements in men [74]. Effect sizes across six studies ranged from 0.26-0.7 with an average of 0.37. For a two-tailed paired-samples t-test measuring free testosterone, with an alpha of 0.05 and 95% power to detect an effect of 0.37, a sample size of 97 is required. Multiplying by 1.3 to account for attrition, we arrive at 126 per group. With two groups the total comes to 252 participants. Thus, our sample of 150 participants per group should be sufficient to detect an effect of this size.

We hypothesize that, along with the decrease in EDC exposure, individuals will exhibit improved health biomarkers after EDC report-back. Additionally, we posit that individuals receiving the treatment (i.e. online curriculum, coaching, and community forum support), will show both increased EDC reduction and improved health biomarker levels compared to the control (report-back only) group.

### Data collection, management, and analysis

The MM *Detect and Detox* kit is an at-home sample collection service that participants will use to collect their own samples. The mailed kit has the following components: kit box, instruction card with activation code, sterile urine collection cup, biohazard bag with absorbent paper, pre-labeled polymailer. We have built a secure online portal for users to make accounts, register their kits, track the status of their samples, and view their testing results and feedback online. For the Lifestyle Audit, participants in the intervention group will schedule a Zoom call with a MM lifestyle coach to document the food, drinks they consumed, as well as all the products (personal care and household products) they used on the day prior to urine collection. MM’s technology team will oversee the security of MM’s software stack and servers, ensure that the platform is HIPAA-compliant, and oversee the software building and maintenance for this project.

Several emails will be sent to the participants to update them on their sample and report statuses and keep them engaged. FedEx Priority Overnight shipping will be used to ensure the sample quality. The turnaround time for the lab analysis and MM report generation is 2-4 weeks. Three months after submitting their baseline urine sample, participants will be sent the second kit and go through the same process again and submit their second samples. Detailed flow of events is illustrated in Fig. 1.

A third party, internationally well-known state-of-the-art laboratory is in contract with MM to perform the sample analysis. The lab staff will be blinded to participants’ identifiable information to ensure no bias will be introduced during sample analysis. The following common EDCs [75] are included in the panel: bisphenol A (BPA), detected in 95% of NHANES participants; [5] bisphenol S (BPS) and bisphenol F (BPF), detected in 89% of NHANES participants; [21]; low molecular weight phthalates, including mono-ethyl phthalate (MEP) and mono-n-butyl phthalate (MBP), metabolites of diethyl phthalate (DEP) and dibutyl phthalate (DBP); high molecular weight phthalates, including, mono(1-ethylhexyl) phthalate (MEHP), mono(2-ethyl-5-carboxypentyl) phthalate (MECPP) and mono(2-ethyl-5-hydroxyhexyl) phthalate (MEHHP) metabolites of di-(2-ethylhexyl) phthalate (DEHP) detected in over 90% of NHANES participants; [22] methyl paraben, ethyl paraben, propyl paraben, and butyl paraben, found in 90% of NHANES participants, [76] and benzophenone-3 (BzP-3), detected in 97% of NHANES participants [62].

We will analyze urine samples by Liquid Chromatography Mass Spectrometry (LCMS) for the presence of the target metabolites listed above. All targets have commercially available isotopically labeled standards, called “surrogates.” The isotopically labeled surrogates will be spiked into the urine before sample preparation. The level of the surrogates will be determined during method verification. Sample preparation will consist of solid phase extraction per the protocol established during method verification. A solvent blank will be prepared concurrently. An internal standard will be spiked into each sample and blank after extraction. Native target response, if detected, will be reported as a ratio to the isotopically labeled surrogate. Two replicates will be run per sample to ensure reproducibility and repeatability as part of the quality control and assurance. Results will be reported to MM including sample ID, date received, date analyzed, metabolite levels, limit of quantification and detection, as well as specific gravity (to adjust for individual hydration status).

After analyzing the samples, we will calculate the percentiles (25^th^, 50^th^, 75^th^ and 95^th^) of analyzed samples and compare participants’ levels for each of the metabolites with existing MM users, as well as the national averages derived from NHANES. Participants’ metabolite levels will be categorized into low (below 25^th^ percentile), medium (equal or above 25^th^ to 75^th^), high (equal or above 75^th^ to 95^th^), and very high (equal or above 95^th^). For the intervention group, MM will also audit their lifestyle behaviors and products used. Problematic areas, such as handling thermal receipts, consuming canned food or drinks, and using products with fragrance, the presence of other harmful ingredients, etc. will be listed in the report along with the lab results and comparisons. The MM team will also be blinded to participants' identifiable information to ensure no bias is introduced. A detailed list of personalized behavior and product recommendations will be provided to the participants for them to follow and make changes. The control group will receive generalized recommendations, based on their exposure levels, that cover multiple areas, including diet, home environment, personal and household products, etc. The same process will be performed for the second kits. EDC levels from participants at baseline (first test) and second test will be compared, and factors such as food, diet, product use, health status, and stress and physical activity levels will be correlated with changes in EDC exposures. Covariates, including sex, BMI, age, education level, household income, and social support will be included in the analysis.

For the analysis plan, we will examine the distribution and concentrations of each of 13 chemical analytes in urine to determine geometric means, medians, percentiles and ranges of exposures. We will compare the percentiles with those reported in the NHANES study. We will include BMI, age, gender, diet, education and household income as covariates. Participants’ dietary patterns will be captured prior to sample collection by looking at their consumption of packaged food and times eating out as these are important culprits of EDC exposures. We will conduct an intent-to-treat analysis. The primary outcomes are the pre-post (first and second tests) differences in specific gravity-adjusted concentration levels per analyte. Clinical biomarkers measured before and after the intervention will also be compared to assess improvement in health and reduced disease risk. We willassess changes in participants’ EHL, RtC and well-being before and after the interventions as well. We will first generate summary statistics on participants’ responses. Changes will be calculated based on paired values of individual responses.

For all analyses, data will be reviewed to ensure assumptions are not violated. If outcomes are not sufficiently normally distributed for parametric tests to be valid, nonparametric tests will be conducted. If needed, variables with response categories with too few participants will be combined. Attrition analyses will be run to compare demographic variables between participants who completed post-test surveys and those who did not.

Summary data and associations between demographic variables and EHL, RtC, well being, EDC metabolites, and clinical biomarkers will be analyzed for all participants with pre-test survey data. Independent sample t-tests, one-way ANOVAs, Chi-square tests, and Spearman’s rank correlations will be run as appropriate. Changes from pre-test to post-test comparing differences between the intervention and control groups, will be analyzed for participants who completed both surveys using regression analyses, controlling for significant demographic variables.

We have established a data monitoring committee for this study, which includes statisticians, chemists, and epidemiologists. Ongoing (weekly) project oversight will occur by the project PI (Dr. Hua) in conjunction with Dr. Rochester. In addition, our MM’s Chief Technology Officer, who has extensive experience in the fields of internet security, computer science, and HIPAA regulations—will participate in the project’s Data and Safety Oversight Committee, which will meet at regular intervals (e.g., semi-annually or more frequently as indicated) throughout the project period to provide input and feedback related to study recruitment, data completion rates, measurement issues, and adverse events*. *MM and HNP will have access to the final trial dataset. Personal information from enrolled participants will be stored separately from results to protect confidentiality, and deidentified data will be shared with participants after the trial.

### Expected outcomes and limitations

We expect participants to have significant reduction in EDC levels after the report-back and intervention with a greater effect in the intervention group. We may experience a bias towards the null if users are actively trying (or begin trying) to reduce EDC exposures at the start of the study. This potential issue will be addressed by examining the EHL and readiness to change surveys, to assess any previous or ongoing EDC reduction activity. In previous work, EHL was high in participants at baseline [44]. This was due to the insensitivity of the survey for EDC-specific EHL. We have since modified our EHL survey to specifically assess EDC EHL knowledge, attitudes, and behaviors, and will utilize this modified EHL survey in the current proposal. In terms of quality control of lab analyses, LCMS can have technical issues known as batch effects and inefficient compound identification. We will minimize batch effects by taking several cautions: 1) samples will be collected as described in a Standard Operational Protocol and stored at -80^o^C until analysis; 2) all samples will be analyzed in a single batch for each LCMS setup to eliminate batch variation; 3) standard mixture of known benchmark metabolites will be analyzed within each batch at multiple time points to control for fluctuation in instrument performance; and 4) technical blank samples are included periodically throughout sample collection to control for variation in sampling. To overcome identification inefficiency, we will use post-acquisition strategies such as running available standards and representative samples to identify unannotated metabolites that are differentially expressed over time with high confidence. The resulting elution time and mass fragmentation pattern will be used as evidence of the potential chemical identity of unannotated metabolites. The analytical laboratory will also provide additional analysis data including target retention time (RT), target response, surrogate RT, surrogate response, surrogate RT, surrogate concentration, internal standard RT, internal standard response, date received and analyzed for potential troubleshooting. If any kits/samples are lost in transit, participants will be sent a new kit. The study team will communicate on a regular basis to ensure any problems that arise will be addressed in time, and the study will be conducted with the highest rigor. The biomarker assessment is exploratory, and it is possible we will face unexpected challenges, such as difficulty recruiting people to take the blood test. We will address these issues as they arise, adjusting our approach as needed.

### Monitoring

Trial conduct will be closely monitored by the MM and HNP teams to ensure strict compliance with data management and promote study quality. We do not anticipate any risks to the subjects, but any adverse events will be noted.

## Discussion

This study, which builds on our previous work, will demonstrate the effectiveness of the Million Marker endocrine disrupting chemical (EDC) exposure report-back on increased EDC environmental health literacy (EHL), readiness to reduce exposures (i.e. readiness to change, RtC), and exposure reduction itself, in EDC-tested individuals. Additionally, we hypothesize that providing further intervention beyond report-back (i.e. EDC-specific environmental health literacy education and community support) will be a measurable improvement over report-back alone, in terms of EDC EHL and exposure reduction behavior, a reduction in individual exposures, and an improvement in clinical biomarkers.

### The need for EDC exposure reduction intervention

In order to mitigate the health impacts and corresponding financial burden of exposures to EDCs, individuals must have access to an affordable, easy, and rapid way to test their exposures to EDCs. Additionally, improving the environmental health literacy (EHL) of individuals is imperative for them to understand *how* to reduce these exposures. Environmental health literacy (EHL) is the knowledge of harmful environmental exposures and how they affect health [66, 77, 78]. Although environmental exposure (exposome) contributes 70% to chronic disease risk, [79] EHL is low in the general population [77] and among healthcare providers [80–82]. To address this, the National Institutes of Health (NIH) has called for increased EHL research, including methods to increase EHL, [77] and for applications (RFAs) of EHL research [83]. Thus, the Million Marker (MM) EDC testing kit and service is a major step in allowing the public to “learn what’s inside” of them and take action to reduce their personal exposures. The MM test kit is a non-invasive (i.e. urine), affordable, and scalable personalized environmental exposure testing and analysis service, followed by a tailored intervention program to empower individuals to optimize their health, prevent diseases, and manage existing conditions.

### Previous work and current protocol

In previous work, we developed and tested the first mobile EDC intervention service (exposure report-back and personalized recommendations via mobile app) for its efficacy in reducing EDC levels, increasing EHL, increasing readiness to reduce exposures (i.e. readiness to change, RtC), and assessing system usability among reproductive-age participants recruited from the Healthy Nevada Project (HNP), one of the largest population health cohorts in the world. We found decreased EDC exposure with the intervention, as well as increased RtC in women (but, interestingly, a decrease in RtC in men) [44]. However, participants had difficulty carrying out the intervention on their own. Because of the difficulty in the EHL subject matter, participants still felt ill-prepared to apply their knowledge to making healthier lifestyle changes and cited financial reasons and limited choices as barriers to change [44]. Therefore, in the current study, we will address these perceived limitations by testing a self-directed online interactive curriculum with live counseling sessions and individualized support modeled after the highly effective Diabetes Prevention Program (DPP) [84] and Omada Health [63] (which provides a digital interactive DPP).

The at-home Million Marker testing and report-back service, along with the online curriculum, will provide an easy-to-use roadmap for individuals and communities to reduce personal exposures and optimize health. Additionally, it allows for scalable, economical, and far-reaching exposomic population research and intervention studies, with opportunities for industry, academic, and government partnerships.

### Trial status

Recruitment of HNP participants to this study beganJuly 2024, with subjects being recruited into study waves with staggered starts for ease of recruitment and data collection. The initiation of study events began as the first wave of participants was recruited, September 2024.

## Supplementary Information

Supplementary Material 1.

Supplementary Material 2.

## Data Availability

Full protocol and data will be available upon request from the authors. Results of this study will be published in peer-reviewed journals, with MM and HNP as authors. We will comply with the clinical trial information dissemination expectations of the NIH policy to register and submit summary results at ClinicalTrials.gov, updating data and information at least once per year. Any apparent errors, deficiencies, and/or inconsistencies identified by NIH as part of the quality control review process and any other errors identified will be addressed by the responsible party. Trial results will be submitted no later than one year after the primary completion date. Informed Consent Documents for the clinical trial will include a specific statement relating to posting of clinical trial information at ClinicalTrials.gov. Once data collection is complete, MM’s statistician will prepare and submit trial results no later than one year after the primary completion date.

## Abbreviations

Apo-AI: Apolipoprotein AI
ApoB: Apolipoprotein B
BPA: Bisphenol A
BPF: Bisphenol F
BPS: Bisphenol S
BUP: Butylparaben
DHEA: Dehydroepiandrosterone
DPP: Diabetes Prevention Program
EDC: Endocrine disrupting chemical
EHL: Environmental health literacy
EPB: Ethylparaben
FSH: Follicle-stimulating hormone
HbA1c: Hemoglobin A1C
HDL: High-density lipoprotein
hs-CRP: High-sensitivity C-reactive protein
LDL: Low-density lipoprotein
LH: Luteinizing hormone
MBP: Monobutyl phthalate
MECPP: Mono-(2-ethyl-5-carboxypentyl) phthalate
MEHHP: Mono-(2-ethyl-5-hydroxyhexyl) phthalate
MEHP: Mono(EthylHexyl) phthalate
MEP: Monoethyl phthalate
MePB: Methylparaben
OBZ: Oxybenzone
PPB: Propylparaben
RtC: Readiness to change
SHBG: Sex-hormone binding globulin
TG: Triglycerides
TSH: Thyroid-stimulating hormone
