# Supplementary material for: Reducing Exposures to Endocrine Disruptors (REED) study, a personalized at-home intervention program to reduce exposure to endocrine disrupting chemicals among a child-bearing age cohort: study protocol for a randomized controlled trial
Source: Trials. 2024 Nov 25;25:793. doi: 10.1186/s13063-024-08627-3 (PMC11587698; doi:10.1186/s13063-024-08627-3)
Supplement: Supplementary file 1 — Supplementary Material 1. [file 13063_2024_8627_MOESM1_ESM.pdf]

Research Consent

Title of Study: Renown Institute for Health Innovation-Million Marker Reducing Exposure to Endocrine Disruptors (REED) Study
Principle Investigator: Joseph Grzymski, PhD
Co-Investigator: Jenna Hua
Study Contact: Savanna Grime
Study ID Number: 8012101521
Sponsor: Renown Institute for Health Innovation and Million Marker

Introduction

You are being invited to participate in a research study conducted by the Renown Institute for Health Innovation (RIHI), the sponsor of the Healthy Nevada Project (HNP), in collaboration with Million Marker (MM), an environmental and consumer health company. Before you agree to be in the study, read this form carefully. It explains why we are doing the study; what you must do to participate in the study; what personal information, including genetic information and protected health information, you agree to share as a participant in the study, and other important information you need to know.

At any time, you may ask us to explain anything about the study that you do not understand.

You do not have to participate in this study; your participation is voluntary. If you agree now but change your mind, you will need to contact us to withdraw. At that time, we can explain to you what happens with the information you have already shared with us.

This study will be registered at ClinicalTrials.gov.

Why are we doing this study?

The overall goal of this study is to improve the population health of Nevada and beyond. To do so, we are obtaining environmental exposure, clinical biomarker, and lifestyle data from Nevada residents to combine with some of your current coded (names and other identifying information has been removed from the data and replaced with a code) data from the Healthy Nevada Project, with the goal of understanding factors that may be related to endocrine-disrupting compound exposure. The data we collect from this study will be made anonymous by coding and storing them in an exposure/health information database. The data will be used to look for patterns and other statistically relevant information that may be beneficial in predicting, planning for, and positively influencing the health, health decisions and health care needs of Nevada citizens. We will also be studying how environmental exposure insights influence the health and lifestyle decisions made by study participants.

In addition, the coded data in the exposure/health information database may be made available for other research projects and to researchers outside of RIHI, as approved by RIHI, including for commercial or for-profit purposes. For example, the coded data will be available to researchers who are seeking to understand how environmental exposures are related to certain diseases, development of new scientific methods, and development of new health recommendations. This is just one example of how coded data may be used. MM and RIHI will vet and approve all requests from outside researchers before providing them with access to coded data.

From time to time, researchers from MM-RIHI or other institutions might want to ask you to participate in additional research studies or development projects. In some cases, you might be a particularly good candidate for a particular study because of your environmental exposures, location, or health history. By signing this document, you give your permission for MM-RIHI to contact you about future research opportunities. The information that MM-RIHI will use to contact you is stored separately from the coded exposure/health information database. You may withdraw your permission to be contacted about future research at any time by contacting the Principal Investigator of the study.

Why are we asking you to be in this study?

We are asking you to consider being a part of this study because you are a resident of Nevada or surrounding areas and you indicated to us your interest and willingness to participate in future research opportunities when you joined the Healthy Nevada Project.

How many people will be in this study?

We expect to enroll up to 600 participants from across the geographic area of Northern Nevada in this phase of our study.

What will you be asked to do if you agree to be in the study?

If you decide to enroll in this study, your participation will entail:

- Completion of surveys asking about your knowledge and desire to reduce exposure to harmful environmental chemicals, as well as your general health, physical activity, stress, sleep, and lifestyle habits. In addition, at the conclusion of the study, you will be asked to complete a survey of your opinions about the study.
- Be randomized (assigned by chance) into one of the study groups
- Submission of two urine samples
- Submission of two blood samples (optional)

You may also be asked to participate in activities aimed at educating you about environmental health, and gathering information about your lifestyle and health.These include:

- Participation in two lifestyle audits documenting your diet and product usage 24 hours prior to each urine sample

- Viewing of up to eight video educational sessions
- Participation in one-on-one sessions with coaches, group discussions with coaches, and/or online forums aimed at reviewing educational course content and answering your questions

As part of the process, you will receive a urine sample collection kit and an optional blood sample collection kit during the first week of the study, followed by a second urine and optional blood kit in the last week. Each kit will include instructions on how to collect, package, and mail-in your samples.

Million Marker, our research partner, will utilize the urine samples to assess your exposure to common toxic chemicals found in various aspects of daily life—such as foods, drinks, personal and household products, and ambient air and dust. Through the Liquid chromatography-mass spectrometry (LC-MS) process, Million Marker will identify biomarkers in your urine, which represent chemicals processed and eliminated by your body. Additionally, your lifestyle audit, documenting your diet and product usage 24 hours prior to each urine sample, will aid in pinpointing the sources of identified exposures. Siphox Health, our clinical lab collaborator, will analyze your blood samples to assess a wide range of biomarkers involved in cardiovascular diseases and inflammation, metabolic disease and diabetes, hormones and fertility, stress, and cancer.

Following the urine and blood analyses, you will receive a comprehensive report outlining your exposures, potential sources, and recommendations to minimize environmental chemical exposures, as well as a report detailing levels of clinical biomarkers tested. After approximately 10 weeks, you will be sent the second urine kit, and may be asked to participate in another lifestyle audit. You will also be sent the second blood sample kit. After returning the urine and blood samples, detailed urine and blood analysis reports will be provided, guiding you on steps to reduce your environmental chemical exposures based on the updated information.

If you agree to be in this study, you agree to the following:

- You authorize MM to share your exposure information with RIHI and with MM and its partners who perform additional analyses.
- You authorize MM to store your coded information and leftover urine samples for future research and product development. We will remove your name and other identifiers from your sample and data, and replace them with a random number. We will keep the list that links the random number to your name separate from your sample and data.
- For the duration of the study you will be periodically contacted via email or web survey by RIHI and MM and asked to answer questions that will provide additional important information about you that will be used as part of the research.

What do you have to do to be in the study?

To be in this study, you must be an existing Healthy Nevada Project participant between 18 to 44 years of age and in good health, not pregnant, free from diabetes or known kidney disease or cancers. You must also be able to understand written and spoken English, be willing to complete all study assessments, and not have participated in the related previous pilot study.

Once you have fully read this document and chosen to join the study by signing our electronic consent, you will be randomized (assigned by chance, like a flip of a coin) into one of the study groups. Neither you nor the researchers choose your assigned group. You will have an equal chance of being in either group.

Next, you will be sent a survey by email and a test kit will be mailed to your home at the address you provide in your completed consent. When you receive your kit in the mail, depending on which study group you were randomized into, you may or may not be provided with a link to schedule a call with a Million Marker Coach to participate in a lifestyle audit. During this call, you will be asked about your 24-hour diet and product use, and the call should take 45-60 minutes. The following morning, you will need to follow the enclosed kit instructions to collect a urine sample. The urine sample should be collected the first time you urinate in the morning and returned as soon as possible in the prepaid return mailer to your nearest FedEx location anytime Monday through Thursday. After your sample has been received, it will be analyzed and your results will be sent to you as a PDF report. Million Marker will provide you with recommendations for reducing your exposure to harmful chemicals that you may choose to implement into your lifestyle.

If you choose to participate in the optional clinical biomarker add-on study, you will also receive a test kit from Siphox Health to your home address. When you receive your kit in the mail, you will need to follow the enclosed kit instructions to collect blood using the included blood collection card via finger pricks. This sample should be returned as soon as possible in the prepaid return mailer using the US Postal Service (USPS). After your sample has been received, it will be analyzed and your results will be sent to you as a PDF report.

Approximately 10 weeks after your first urine sample, you will be mailed a second sample kit to your home. You will again be asked to provide a urine sample the following morning, and return the sample as before. You may also be asked to participate in a lifestyle audit. Million Marker will analyze your second urine sample and return the results to you from the analysis. At this time, you will also receive a second Siphox Health test kit if you choose to participate in the optional add-on study and you will again be asked to provide a blood sample and to return the sample as before.

For the duration of this research study, you will be periodically contacted via email to provide you with resources on environmental health and tips on reducing exposure to harmful chemicals and asked to answer several questions providing additional relevant information about you. You

may also be periodically contacted via email with important information, such as new research developments, insights or opportunities being offered to participants.

How long will you be in the study?

The duration of the study is approximately 6 months from the first to last survey you complete. After that, you will receive monthly emails from MM with resources on environmental health and tips on reducing exposure. You may unsubscribe from these emails at any time.

Your coded exposure and health information will be stored indefinitely in a database, and your urine samples stored in a biobank, for future research use, as described under “Why are we doing this study?” In addition, you may be contacted regarding future research opportunities unless you withdraw your permission.

What happens if you choose not to be in this research study?

If you decide not to be in the study, you will not be asked to provide a urine sample, complete surveys, or participate in any of the activities listed above, and there is no other obligation.

What if you agree to be in the study now, but change your mind later?

You may withdraw from the study at any time by notifying the Principal Investigator of the study. The result of withdrawing from the study is that you will no longer receive any emails or other communication as part of the study. Your coded exposure information will continue to be a part of the exposure/health information database and will continue to be used by the researchers and may be used for future research.

What are the risks associated with being in this research study?

Your participation in this study is non-invasive and there is no risk from providing a urine sample. However, there are risks involved in having your exposure information analyzed and in sharing your exposure information.

- Your exposure data may reveal that you have high exposure to certain chemicals, such as endocrine disrupting chemicals (EDCs), which may negatively impact your health. You may choose to seek additional healthcare advice or treatment, however any medical advice or treatment that you seek based on your results will be your financial responsibility.
- As with any database, despite RIHI and MM implementing rigorous privacy and security measures to protect the privacy of your information, there is always a chance that your exposure data, health information, survey responses, and/or personally identifying information may be stolen in the event of a security breach.

Although RIHI and MM cannot provide a 100% guarantee that your data will be safe, they have strong policies and procedures in place to minimize the possibility of a breach. In addition to the risks noted above, there may be additional risks to participation that are currently unforeseeable.

What are the possible benefits from participating in this research study?

As a population health study, the primary purpose of this research study is to help researchers and clinicians better understand how exposure information may be used to improve the health of individuals and communities. Although the study has not been designed to impact your immediate clinical care, the practice of exposomic medicine (which means medical care and clinical decision making that is informed by exposure information) is evolving to make a positive impact on an individual level. There is a small chance that the biomarkers provided to RIHI could also reveal information about you that is important for your health. Learning about these risks can be valuable so that you and your healthcare providers can make informed decisions about what next steps are most appropriate for you.

By participating in this study, you will receive information about your environmental exposures and personalized lifestyle recommendations from MM. More information about MM and MM test results will be provided to you during the registration process. If you do not want to receive the results, you should not participate in this study.

When you enroll, the study will provide information on which insights you can expect to receive soon after providing your sample for exposure analysis. As the study evolves, it is possible that other results will be made available to you.

As with any voluntary research study, you may withdraw at any time by contacting the Principal Investigator of the study.

Who will pay for the costs of your participation in this research study?

There is no cost to you associated with participation in this study, including the cost of the exposure analysis provided by MM and RIHI. If the analysis of your exposures reveals important health information and you choose to share the information with your healthcare providers, further testing to confirm your results may be recommended. The cost of this additional testing will be your responsibility through personal payment. Additionally, any medical advice or treatment that you seek as a result of that information will be your responsibility.

Will you be paid for being in this study?

You will receive insights about your exposure and lifestyle recommendations at no cost to you (the two urine test kits and associated reports are valued at $598). In addition, you will receive gift cards after answering surveys. There will be 3 surveys for a total of $75 in gift cards.

If your information is used as part of or to create valuable products or services, there are no plans to pay you or give any compensation to you and your family.

Who will know that you are in this study and who will have access to the information we collect about you?

The researchers who conduct the statistical analyses do not have access to Registration Information (name, address, and email address) of participants but do have access to your coded information, which is assigned a unique code in order to protect your identity.

Research staff who interact with research participants have access to names and contact information of participants, but no laboratory data or survey information. All research staff are trained on how to work with human research participants. In addition, all researchers are trained on how to conduct research responsibly. MM will have access to the information you provide to MM during the lifestyle audit and any subsequent data you provide to them. The research team at MM may analyze your data, after it has been coded, for research purposes and share the coded data with RIHI. RIHI may also provide your coded data to other researchers conducting approved and vetted research projects in the future, as described under “Why are we doing this study?”

RIHI will use your registration information to contact you about future research opportunities or to provide you with information that we find is important for your health. Your registration information will never be associated with your coded data in the exposure/health information database that will be used for research purposes.

How will we protect your private information and the information we collect about you?

We will treat your identity with professional standards of confidentiality and protect your private information to the extent allowed by law. RIHI and MM have strong data privacy and security policies and procedures in place to protect your information and minimize the possibility of a data breach.

During the initial phases of the study your coded exposure data will be provided to researchers. The coded data may be associated with other coded data in the RIHI database using unique codes/identifiers. If other researchers request access to your data for use in future research, we

will only provide your coded data. Your name will not be used in any publications or reports that result from the study.

At the end of this consent form, you will be asked to provide us with your contact information. We will share your contact information with MM for the limited purpose of sending you a urine collection kit, and ensuring the kit is appropriately tracked in MM's systems, as well as sending you engagement emails throughout the study. MM will protect your information using the methods and practices stated in MM's Privacy Policy, Terms of Use, and Platform Consent, which you can review by clicking the links or visiting millionmarker.com. You can also review Siphox's Privacy Policy and Terms of Service.

Do the researchers have monetary interests tied to this study?

This study is being conducted by Renown Institute for Health Innovation and Million Marker, a private company, as a study to demonstrate the ability to obtain important exposome data using urine samples. Results from this study may be used to apply for future funding through research grants or sponsor funded studies. As such, each entity may benefit financially if future funding is secured, however, researchers' direct compensation is not dependent on the outcomes of this research.

This disclosure is made so you can decide if this relationship will affect your willingness to participate in this study. Renown Institute for Health Innovation is providing their scientific expertise and research staff to conduct this study. Million Marker is contributing the urine kits, data collection via phone app, results, and staff resources, including Jenna Hua's salary, to conduct this study. Ms. Hua is the Founder and CEO of Million Marker and is a member of the research study only involved in aggregated data analysis and research publications as a result of the study.

Who can you contact if you have questions about the study or to opt-out?

At any time, if you have questions about this study, contact Joseph Grzymski, PhD (Principal Investigator) xxx-xxx-xxxx; or Savanna Grime (study coordinator) xxx-xx-xxxx.

Agreement to be in study

We will give you a copy of this form to keep.

By signing your name below, you agree to be in this study and acknowledge and agree to the following:

1. You acknowledge that you have been given the opportunity to fully read this form and ask any questions.

2. You agree to fully participate in the registration and education process to participate in the study.
3. You agree to participate in the study by reasonably completing the steps outlined in this consent document.
4. You authorize Million Marker to analyze your exposures from your urine samples, securely store that information indefinitely, and share your exposure information with the RIHI researchers conducting the study as described above.
5. You authorize Siphox Health to analyze your biomarkers from your blood samples, securely store that information indefinitely, and share your exposure information with the RIHI researchers conducting the study as described above.
6. You authorize that your age, ethnicity, address, email, phone number and coded survey and exposure information may be used as part of the database for the study, which will be maintained and used for future research by RIHI and other researchers with the approval of RIHI.
7. You agree to participate in the study by reasonably responding to email and survey requests for additional data and allow such additional data to be used in the study.
8. You agree to receive exposure-related insights from the study that may enable you to take action and make informed decisions based on your results.
9. You agree that RIHI may contact you about future research opportunities that may arise from your participation in this study.
10. All rights and obligations herein may be transferred by RIHI to any successor organization.

____

Participant’s Name Printed

______________________________________ ________________________

Signature of Participant **Date**

Authorization to Release Health Information that Identifies You for a Research Study

If you sign this document, you give permission to researchers at RIHI to release health information that identifies you for the research study described in this consent form.

The health information that we may release for this research includes your:

- Name
- Email address
- Phone number
- Mailing address

The health information listed above may be released to **Million Marker** for the limited purpose of shipping urine collection kits to you and ensuring the kits are appropriately tracked in Million Marker's systems, as well as sending engagement emails throughout the study.

I may revoke this Authorization at any time, in a written revocation sent to the principal investigator or Custodian of Records. However, I understand that my health information might have already been released.

Information released by this Authorization might be re-disclosed by the recipient and might not be protected by state and federal privacy laws. I agree to release Renown Health from liability for release and disclosure of the released information. RIHI is required by law to protect your health information. By signing this document, you authorize RIHI to release your health information for this research.

Please note that:

- You do not have to sign this Authorization, but if you do not, you may not participate in the study.
- RIHI may not condition treatment on whether you sign this Authorization.
- You may change your mind and revoke this Authorization at any time, except to the extent that RIHI has already acted based on this Authorization.
- This Authorization will expire in ten years.
- A copy of this signed Authorization will be sent to you.

CONTACT INFORMATION

Email Address: ____

Date of Birth: ____

Home Phone: ____

Mobile Phone: ____

Address: ____

Address (cont.): ____

City:________________ **State:**________________ **Zip Code:** ________________

Ethnicity: ____

Birth Sex: ____

___ **I am at least 18 years old**

____ ____________________________

Signature of Participant **Date**
