# Supplementary material for: Reducing Exposures to Endocrine Disruptors (REED) study, a personalized at-home intervention program to reduce exposure to endocrine disrupting chemicals among a child-bearing age cohort: study protocol for a randomized controlled trial
Source: Trials. 2024 Nov 25;25:793. doi: 10.1186/s13063-024-08627-3 (PMC11587698; doi:10.1186/s13063-024-08627-3)
Supplement: Supplementary file 2 — Supplementary Material 2. [file 13063_2024_8627_MOESM2_ESM.docx]

**Statistical Design, Power and Analysis Plan**

Table 1 illustrates the power to detect an effect size of each of our 13 endpoints, from a sample of n = 150 participants per arm (i.e., after 50% attrition down from the original n = 300 per arm). We see that our sample is likely best-powered to detect statistically discernible differences in pre-post changes between treatment arms for the WHO-5 (99.8% power) and BPA (97.5% power) endpoints.

**Background.** (See the study protocol for references. Additional references are listed under References.)

Phase I was a pre-post single-arm intervention study with post-intervention data collected after three days. Building on our Phase I progress, In this Phase II proposal, we will continue our product development and testing by 1) developing and 2) testing a self-directed online interactive curriculum with live counseling sessions and individualized support modeled after the Diabetes Prevention Program (DPP)[^20^](https://www.zotero.org/google-docs/?2JYA51) and Omada Health[^21^](https://www.zotero.org/google-docs/?OaUbLF) (which provides a digital interactive DPP). By developing, testing and improving this new intervention option, we will be one step closer to product-market fit and offer a cost-effective service for reducing EDC exposures, increasing EHL/RtC, and improving clinical and well-being outcomes.

We **hypothesize** that the self-directed online interactive curriculum with personalized support will be more effective (superior) than the mobile EDC reduction program at increasing EDC-specific EHL, readiness to reduce exposures and well-being (**Aim 2**), as well as reducing EDC urinary chemical metabolites (**Aim 3**). These two arms will be tested in a longitudinal EDC randomized control trial (n=600). We will recruit and randomize 600 participants (1:1 male to female) in reproductive age from HNP, our Phase I collaborator. Outcomes for this aim will measure surveyed changes pre- post-interventions in EDC EHL, EDC-specific knowledge/behavior/attitudes, RtC, and well-being. We will utilize the same validated instruments used in Phase I and WHO well-being scale[^23^](https://www.zotero.org/google-docs/?nww06G) to assess these outcomes.

Using our existing EDC panel (used in Phase I, part of MM’s current direct-to-consumer testing service) that tests for 13 chemical metabolites including BPA, BPA alternatives, phthalates, parabens and oxybenzone, participants’ urine samples will be collected twice (at pre- and post-intervention) to measure changes in EDC levels in **Aim 3**. We expect the creatinine-adjusted urinary concentration of these chemicals to be log-normally distributed. Hence, all calculations will be done on log-transformed analyte values, which we will assume are fairly normally distributed.

There are three outcomes of interest in Aim 2, and 13 in Aim 3. The endpoint in each of the following hypotheses is a function of its outcomes. Specifically, the endpoint is the difference between the intervention and control arms (which are randomized) in the mean pre-post change (i.e., from a pre-intervention period to the end of a post-intervention period after three months) of the corresponding outcome.

**Objective.** We have a given sample size of n = 300 per arm for each of two randomized treatment arms. For each of the sixteen total endpoints, our objective is to characterize the possible statistical power we might have to detect a difference of $\delta$ or greater in mean endpoints between arms.

We will assume that continuous endpoints vary with the same standard deviation (SD) $\sigma_{Y}$ in both arms. We will also assume these endpoints are fairly normally distributed, allowing us to rely on the two-sample t-test in our calculations carried out via the R command pwr::pwr.t.test(). For binary endpoints, we will use the R command pwr::pwr.2p2n.test() to calculate the power for a difference in proportions between arms.

We will use a combination of literature references and our own preliminary Phase I findings to posit reasonable values of $\delta$ and $\sigma_{Y}$.

**Approach.** We will proceed as follows, grouped by section.

1. **Hypotheses, Outcomes, and Endpoints**: We will attempt to specify a reasonable a priori $\delta$ and $\sigma_{Y}$ for each endpoint using either the literature or our own preliminary findings.
2. **Adjustments**
   1. **Number of Tests**: We will report the number of a priori statistical hypothesis tests we plan to conduct, and the way we will handle multiple testing.
   2. **Anticipated Attrition**: We will account for anticipated proportion or rate of sample attrition (i.e., loss to follow-up).
   3. **Additional Assumptions**: We will report any additional assumptions needed to proceed with our analysis.
3. **Results, Conclusions, and Recommendations**
   1. For our fixed sample size, we will report the statistical power to detect each endpoint’s effect size.
   2. We will report which endpoint effect sizes our sample is likely best-powered to detect.

**Hypotheses, Outcomes, and Endpoints**

**Aim 2** The three Aim 2 outcomes are the following outcomes, which are survey measures:

- Environmental health literacy (EHL), measured via a validated EHL survey used in Phase I
- Readiness to change (RtC), measured via a validated RtC survey used in Phase I
- Well-being, measured via the five-item WHO-5 Well-Being Index

Our objective is to estimate the effect of our newly developed self-directed online interactive curriculum with live counseling sessions and individualized support on the pre-post change (i.e., change from the pre-intervention period to the end of the post-intervention period) on EHL, RtC, and well-being measured using the EHL, RtC, and WHO-5 Well-Being Index questionnaires.

The EHL measure is a ten-item measure that assesses environmental health literacy.^110^ The Phase I RtC measure is based on the original American Council on Exercise RtC Questionarre.^111^ It is a four-level ordinal measure that assesses readiness to change. For our calculations, we will dichotomize these four levels into “yes, I am already in the process of reducing my exposure to harmful chemicals” versus otherwise. The WHO-5 Well-Being Index is a five-item measure that assesses well-being.^23,109^

For all measures, the item scores range from 1-5, producing total scores from 5 to 50, with higher scores indicating a better outcome. Hence, the minimum possible pre-post change for a given participant is -45, and the maximum is 45.

For our mainly exploratory study, we seek to characterize what constitutes a minimum clinically important difference (MCID), either positive or negative, in the mean pre-post change between treatment arms for each measure. Hence, in order to characterize sample size and power, we will use the following values as the mean changes to expect in the control arm (i.e., not the main study endpoint, which is the difference in mean changes between the control and treatment arms), along with their standard deviations (SDs) where available. The Phase I values are interim findings; the study is still ongoing.

- Our Phase I preliminary estimate of a mean pre-post change in EHL is -0.10, after using Million Marker’s Detect and Detox kit and receiving chemical metabolite test results and personalized recommendations. This spanned roughly 11 weeks of participation on average (see Appendix). Based on an EHL SD of 3.22 at pre-intervention and 3.74 at post-intervention, we will assume the standard deviation of pre-post changes is the average of these values; i.e., SD = 3.48.
- In Phase I, we also estimated a pre-post change of 0.098 (i.e., 36/80 - 31/88 in Appendix Table 1) in the proportion of “yes” RtC responses.
- Castro Sweet et al (2018)^99^ estimated a mean change of 2.72 (SD 4.23) in the WHO-5 Well-Being Index, after 16 weeks of participation in a “program combining digital health with human coaching for diabetes risk reduction.”^99^

For the **analysis plan,** we will assess changes in participants’ environmental health literacy, readiness to change and well-being before and after the interventions. We will first generate summary statistics on participants’ responses. Changes will be calculated based on paired values of individual responses. We will analyze percentage changes by individual responses as well as response categories. A Chi-square goodness of fit test will be used for pre-post comparisons.

**Aim 3** Each Aim 3 outcome is the analyte creatinine-adjusted concentration level, assumed to be log-normally distributed. MEP, MnBP, methyl paraben (MP), ethyl paraben (EP), butyl paraben, propyl paraben (PP), and B-3 pre- and post-intervention values are listed in Tables 2 and 3 in Harley et al (2016),^30^ a pre-post single-arm intervention study with post-intervention data collected after three days. MEHP, MEHHP, BPA, MP, EP, and PP values at pre- and post-intervention are listed in Tables 3 and 4 of Kim et al (2021),^105^ a pre-post two-arm randomized controlled trial with post-intervention data collected after one month (i.e., four weeks).

**Effect Size: Difference between Mean Pre-Post Changes across Arms** Estimated effect sizes and analyte distributions vary in the literature.^87,30,105^ We were able to find representative effect size and SD values for 10 of the original 13 analytes; hence, our calculations are based only on these 10. The three excluded analytes are BPS, BPF, and MECPP.

For MEHP, MEHHP, BPA, MP, EP, and PP, we used the estimates of mean analyte values at pre- and post-intervention in Table 3 of Kim et al (2021) to calculate the estimated mean difference in pre-post changes between intervention and control groups. We used their Table 4 estimates of mean pre-post changes between their intervention and control arms. We privileged the MP, EP, and PP

Kim et al (2021) provided raw post-intervention analyte values, while Harley et al (2016)^30^ did not. We derived the Harley et al (2016) post-intervention values as follows. We first regressed the Harley et al (2016)^30^ Table 2 pre-intervention geometric mean values on their Table 3 counterparts (i.e., a perfect line, as the values followed a deterministic linear adjustment rule that was not disclosed in the text). We then predicted the Table 2 post-intervention values using this fit model.

**Analyte Standard Deviation** We did not find any analyte SDs reported in Harley et al (2016)^30^ or Kim et al (2021).^105^ As a workaround, we approximated the SD for each analyte using its reported pre-intervention geometric mean and 25th, 75th, and 95th percentiles. We did this using the monotonic relationship between the quantiles and log-transformed quantiles for log-normal random variables (<https://en.wikipedia.org/wiki/Log-normal_distribution#Mode,_median,_quantiles>). For MEP, MnBP, butyl paraben, and BP-3, we used Table 2 of Harley et al (2016)^30^ to derive pre-intervention SDs. For MEHP, MEHHP, BPA, MP, EP, and PP, we used Table 3 of Kim et al (2021)^105^ to derive pre-intervention SDs. We assumed these SDs were identical for each analyte at pre- and post-intervention.

Neither Harley et al (2016)^30^ nor Kim et al (2021)^105^ reported the SD of the within-subject differences per analyte (i.e., the SD of the within-subject differences in analyte values from pre-intervention to post-intervention) from their mixed-effects models. Instead, to approximate these we examined Figure S1 in the Harley et al (2016)^30^ supplement (<https://ehp.niehs.nih.gov/doi/suppl/10.1289/ehp.1510514>), and thereby set the within-subject pre-post intraclass correlation coefficient (ICC) to 0. See the Additional Assumptions section to see why this is a conservative approach.

**Adjustments.**

**Number of Tests** We anticipate conducting 13 total a priori statistical hypothesis tests (i.e., three for Aim 2, and 10 for the available Aim 3 analytes) for comparing the mean differences in outcomes from pre-intervention to post-intervention. We chose a familywise Type 1 error rate (i.e., corresponding to the familywise statistical significance level) of 0.1 because our study is exploratory rather than confirmatory. That is, we wish to discover treatment effects related to those reported in the literature, rather than confirm ones inferred from past completed randomized controlled trials. Hence, we set the Bonferroni-corrected statistical significance level to 0.1/13 = 0.0077 for each of the 13 a priori tests.

**Anticipated Attrition** In Phase I, 412 participants consented and responded to the pre-intervention survey. However, only 212 participants returned urine samples after sending urine kits to all 412. Of these, to date at least 138 participants also completed the post-intervention survey, and also provided biomarker data. Hence, the Phase I sample attrition rate is currently (412-138) / 412, or 67%.

However, more participants are expected to complete the study, reducing the final Phase I attrition rate. Phase II will involve better and more frequent participant engagement by the study team. Hence, we anticipate that Phase II may have at most 50% attrition. This is the attrition rate we used in our calculations.

**Additional Assumptions**

1. For all Aim 3 endpoints, we based our sample size and power calculations on differences of a log-transformed variable. However, this difference is sensitive to (i.e., dependent on) the original values. This is because $\delta=log(Y_{post})-log(Y_{pre})=log\left( \frac{Y_{post}}{Y_{pre}} \right)$ does not equal another $\delta'=log(Y_{post}+c)-log(Y_{pre}+c)=log\left( \frac{Y_{post}+c}{Y_{pre}+c} \right)$ for some non-zero constant $c$. Hence, our sample sizes will sufficiently power our study only to the extent that the mean of our study’s pre-intervention analytes resembles those reported in Harley et al (2016) and Kim et al (2021).
2. For all Aim 2 endpoints, and the Aim 3 endpoints MEP, MnBP, butyl paraben, and BP-3, we used the literature estimates of mean pre-post changes as estimates of the true difference between treatment arms. Specifically, we made a key common implicit assumption of the pre-post intervention-arm study design: We assumed that the mean pre-post change on the hypothetical control arm is zero. This assumption implies that the true mean pre-post change is identical to the true mean difference in pre-post changes between the control arm and the observed intervention arm.
3. We assumed all outcome pre-intervention SDs were identical to their post-intervention SDs. (This was explicitly stated above for Aim 3.) We also assumed that the pre-post change was the same SD regardless of arm.
4. We assumed the within-subject pre-post ICC was zero. This is a conservative approach: Setting the ICC to 0 treats our sample as if the pre-intervention and post-intervention participants were two completely independent samples. This assumption will require larger sample sizes than would be needed for a higher ICC. More specifically, for each analyte, we used its derived pre-intervention SD (again, assumed to be identical to the post-intervention SD) and pre-post ICC to define the variance of the change from pre- to post-intervention as $2\sigma^{2}(1-ICC)$, where $\sigma$ is both the pre- and post-intervention SD (which we assumed to be equal, as stated in Assumption 3 above).

**Results, Conclusions, and Recommendations**

**Table 1. Power to Detect Endpoint Effect Size**

| **Reference** | **Intervention Period** | **Outcome** | **Endpoint Effect Size** | **Endpoint SD** | **Power** |
| --- | --- | --- | --- | --- | --- |
| Harley et al (2016) | three days | BP-3 | -0.427 | 2.929 | 0.079 |
| Harley et al (2016) | three days | Butyl paraben | 0.66 | 2.592 | 0.318 |
| Harley et al (2016) | three days | MEP | -0.328 | 1.453 | 0.235 |
| Harley et al (2016) | three days | MnBP | -0.121 | 0.772 | 0.095 |
| Kim et al (2021) | four weeks | BPA | -0.554 | 1.029 | 0.975 |
| Kim et al (2021) | four weeks | EP | -0.285 | 2.45 | 0.048 |
| Kim et al (2021) | four weeks | MEHHP | -0.112 | 0.576 | 0.162 |
| Kim et al (2021) | four weeks | MEHP | -0.153 | 0.774 | 0.167 |
| Kim et al (2021) | four weeks | MP | -0.544 | 2.686 | 0.179 |
| Kim et al (2021) | four weeks | PP | -0.879 | 3.366 | 0.338 |
| Phase I | 12 weeks | EHL | -0.1 | 3.48 | 0.01 |
| Phase I | 12 weeks | RtC | 0.098 | NA | 0.035 |
| Phase I | 12 weeks | WHO-5 | 2.72 | 4.23 | 0.998 |

Table 1 illustrates the power to detect an effect size of each of our 13 endpoints, from a sample of n = 150 participants per arm (i.e., after 50% attrition down from the original n = 300 per arm). We see that our sample is likely best-powered to detect statistically discernible differences in pre-post changes between treatment arms for the WHO-5 (99.8% power) and BPA (97.5% power) endpoints.

Note that for analytes, these effect sizes and SDs are not on the original scale; they are on the log-transformed scale. This means they are effect sizes relative to the pre-intervention value; e.g., the effect size is 1 for an analyte with $Y_{post}=2.72$ and $Y_{pre}=1$, and also for an analyte with $Y_{post}=272$ and $Y_{pre}=100$. (See the Additional Assumptions section for details.) Hence, our power findings will most apply to those of our study analytes with a pre-intervention geometric mean close to those in the references used.

For the **analysis plan**, we will examine the distribution and concentrations of each of eight chemical analytes in urine to determine geometric means, medians, percentiles and ranges of exposures. We expect the creatinine-adjusted urinary concentration of these chemicals to be log-normally distributed. We will compare the geometric means with those reported in the NHANES study. We will include BMI, age, gender, diet, education and household income as **covariates**. Participants’ dietary patterns will be captured in the exposure journal prior to sample collection by looking at their consumption of packaged food and times eating out as these are important culprits of EDC exposures. Other covariates have been collected as part of their participation in the HNP. We will conduct an **intent-to-treat analysis**. The primary outcomes are the mean pre-post (first and second tests) differences in creatinine-adjusted concentration levels per analyte (i.e., the mean of the within-subject differences in analyte values from baseline/first test to post-intervention/ second test).

**Appendix**

**Hypotheses, Outcomes, and Endpoints**

**Aim 2**

**Appendix Table 1**

|  | **Before chemical testing** | **After chemical testing** |
| --- | --- | --- |
| **Environmental Health Literacy** (mean (SD)) | **39.36 (3.22)** | **39.26 (3.74)** |
| **Readiness to Change** (n (%)) |  |  |
| Yes, I am already in the process of reducing my exposure to harmful chemicals | 31 (35%) | 36 (45%) |
| Within the next 30 days, Within the next 6 months, or No, I am not ready to make any changes | 57 (65%) | 44 (55%) |

Both the estimated mean pre-post change in EHL of -0.10 and its SD were based on group-level calculations. That is, -0.10 was simply the average change over all participants at post-intervention minus that at pre-intervention. This is different from the ideal calculation, which would involve first taking the difference in EHL values per participant, and then taking the average of these differences. However, this is not a problem because the ideal procedure is algebraically equivalent to ours. For example, { (a-b) + (c-d) + (e-f) } / 3 = (a+c+e) / 3 - (b+d+f) / 3, where the left side of the equation is the ideal procedure, and the right side is what we did instead.

For a given participant, roughly 11-12 weeks passed on average between the pre-intervention and post-intervention surveys. The breakdown is as follows:

- 1 week (receive kit) +
- 3 weeks (average time to submit urine sample) +
- 4 weeks (return results) +
- 1 week (send 2nd survey) +
- 2 weeks (complete survey)

**Code.** See: <https://github.com/ericjdaza/scatter>
